# Whole-Genome Sequence Analysis to Assess Mutations in Efflux Pumps in *Mycobacterium tuberculosis*: The Influence in Drug Resistance

**DOI:** 10.3390/microorganisms13061306

**Published:** 2025-06-04

**Authors:** Miguel Chimal-Muñoz, Damián E. Pérez-Martínez, Gustavo A. Bermúdez Hernández, Paulina M. Mejía-Ponce, Cuauhtémoc Licona-Cassani, Raquel Muñiz-Salazar, Hilda Montero, Roberto Zenteno-Cuevas

**Affiliations:** 1 Health Sciences Doctoral Program, Instituto de Ciencias de la Salud, Universidad Veracruzana, Veracruz 91190, Mexicodamperez@uv.mx (D.E.P.-M.); 2 Instituto de Salud Pública, Universidad Veracruzana, Av. Luis Castelazo Ayala s/n, A.P. 57 Col. Industrial Animas, Veracruz 91190, Mexico; 3 Escuela de Ingeniería y Ciencias, Tecnológico de Monterrey, Monterrey 64849, Mexicoclicona@tec.mx (C.L.-C.); 4 Red Multidisciplinaria de Investigación en Tuberculosis, Ensenada 22890, Mexico; ramusal@uabc.edu.mx; 5 Division of Integrative Biology, The Institute for Obesity Research, Tecnológico de Monterrey, Monterrey 64849, Mexico; 6 Escuela de Ciencias de la Salud, Universidad Autónoma de Baja California, Ensenada 22890, Mexico

**Keywords:** tuberculosis, diabetes mellitus, resistance, efflux pumps

## Abstract

Efflux pumps are proteins related to the transport of molecules in bacteria, and some of them have been recently reported to be involved in drug resistance (DR) in *Mycobacterium tuberculosis*. In addition, the association with type 2 diabetes mellitus (T2DM) has been considered a factor favoring the development of drug resistance. Therefore, the aim of this study was to characterize, by analysis of *M. tuberculosis* genomes, the variants in efflux pump genes and to determine the level of association with T2DM and DR. Nearly 400 Mtb genomes from individuals with and without T2DM and with and without DR were recovered. Of the 164 efflux pump genes analyzed, 10 lack any variant, while 154 genes presented from 3 to 19 variants. The variant S217P in *mmpL13a* (Rv1145) was the most abundant, found in 98 (25%) isolates. A significant association was observed between 19 variants and DR, and between 20 variants and T2DM (*p* ≤ 0.005). Although preliminary, the results show a tendency for certain variants to appear in tuberculosis isolates from individuals with DR and T2DM, demonstrating the possible influence of the host in the evolution of tuberculosis. Further studies are necessary to confirm the participation of these variants in the efflux pump function in tuberculosis.

## 1. Introduction

With more than 10 million cases and 1.5 million deaths annually, after COVID-19, tuberculosis (TB) remains the infectious disease with the greatest global impact on human health [1]. Human Immunodeficiency Virus and type 2 diabetes mellitus (T2DM) are comorbidities frequently observed in TB, with a significant increase in recent years promoting development of drug-resistant (DR) tuberculosis [1,2]. 

T2DM increases the risk of developing TB three-fold and also promotes reactivation of latent infection, rapid progression from latent to active infection, and increased risk of TB treatment failure, relapse, and death [3,4,5]. In addition, individuals with binomial TB-T2DM have a five-fold increased risk of developing monoresistance and a four-fold increased risk of multidrug-resistant tuberculosis (MDR-TB) [5,6,7,8].

Drug resistance in *Mycobacterium tuberculosis* (Mtb) is not only the result of a mutation in the canonical genes. Recently, new physiological processes have been described as promoting Mtb survival against antimicrobials, including DNA damage repair mechanisms and efflux pumps (EPs) [9,10]. These EPs are mostly transmembrane proteins that transport a wide variety of substrates into and out of Mtb cells. The drug resistance conferred by EPs is mainly the result of dysregulation of gene expression, or alteration of protein function in response to antibiotic treatment, that promotes the extrusion of an important collection of molecules, including antimicrobial compounds, thus maintaining a sub-lethal concentration within the Mtb cell, driving a drug-resistant phenotype [11,12,13].

Efflux pumps are divided into five super families based on sequence homology, protein domain structures and function: (1) the ATP-binding cassette (ABC) superfamily; (2) the major facilitator superfamily (MFS); (3) the multidrug and toxic compound extrusion superfamily (MATE); (4) the resistance-nodulation-cell-division superfamily (RND); and (5) the small multidrug resistance superfamily (SMR). All of these families include pumps that are implicated in resistance to several antibiotics in Mtb [11]. 

Although the population structure of Mtb is monomorphic, (this mean low levels of genome diversity and apparently absence of genetic exchange), it has been demonstrated that the host environment modulates genetic variations, enhancing the evolution and adaptation of Mtb to its environment [14,15,16]. However, it is unclear how T2DM influences the development of Mtb and promotes the increase in DR. It was recently shown that T2DM does not have an impact on the development of new or specific polymorphisms in genes directly related to first-line DR in Mtb [17] but does influence the development of polymorphisms in genes related to DNA damage repair, some of which are related to DR [18]. This study aims to characterize efflux pump gene (EPG) variants in Mtb genomes from patients diagnosed with and without T2DM, and with and without drug resistance, and to determine their association with drug resistance.

## 2. Materials and Methods

### 2.1. M. tuberculosis Genome and Database Construction

A search for Mtb genomes was performed in the following public repositories: GenBank (https://www.ncbi.nlm.nih.gov/genome/, accessed on September 2020), TB Portals-NIH (https://tbportals.niaid.nih.gov/), ENA (https://www.ebi.ac.uk/ena/browser/home), and PATRIC (https://www.bv-brc.org/). Inclusion of the genomes in the analysis was conducted according to the metadata information, considering the following criteria: (1) individuals subjected to testing for T2DM; (2) a detailed description of the genotypic profile of sensibility condition and resistance to first-line drugs (rifampicin, isoniazid, pyrazinamide, and ethambutol), considering resistance to only one of the first-line drugs (Mono-TB), poly-resistant (Poly-TB), resistant to two or more drugs except for INH and RIF, and multidrug-resistant (MDR-TB), with simultaneous resistance to INH and RIF; (3) coverage values > 99% and depth > 100×; and (4) belonging exclusively to L4, due to its global distribution and its lack of inherent tendency to develop resistance [19]. Based on host characteristics, the genomes were organized into four groups: (1) individuals with sensitive TB, (2) with drug-resistant TB, (3) with sensitive TB and T2DM, and (4) with drug-resistant TB and T2DM. 

### 2.2. Bioinformatics Analysis

Genome sequences were analyzed following Perez Martinez et al [18]. Low-quality ends in the sequences (<30) were trimmed using Fastp [20]; then, Kraken V.2 [21] was used to filter reads and to avoid false variants as a result of DNA contamination. The reads were aligned with the BWA program using Mtb H37Rv as a reference sequence (Gen Bank ID: ASM19595v2) and considering the default parameters [19]. Variant calling (SNPs and INDELS) was performed following a previously described and validated pipeline [22]. Variants present in at least 20 reads and at ≥90% frequency within each isolate were used to detect phylogenetic mutations and confirm belonging to L4. The L4 sublineages were identified by matching fixed SNPs (those with a frequency ≥90%) to specific phylogenetic positions, as described in a previous report [23]. Those variants in at least 10 reads with a frequency of >10% to <90% were termed non-fixed SNPs and used to predict the first- and second-line drug-resistance profile. Those variants with an allelic frequency >10% in the coding regions of the 175 related efflux pump genes were selected (Supplementary Table S1), and a database was then constructed including the resistance/sensitivity profiles and the presence/absence of DMT2 in the host.

### 2.3. Statistical Analysis and Identification of Specific Variations in the Groups

Inter-group variant differences were analyzed by the Fisher exact test using IBM SPSS V21 [24] (95% confidence level), excluding those SNPs strongly related to sublineages and those present in >99% of the sample. A *p* value of <0.05 was considered statistically significant.

## 3. Results

### 3.1. Characteristics of the Genomes Included in This Study

From the databases consulted, 399 Mtb genomes were recovered; these were mostly from ten countries, including Georgia (33.6%), Moldova (13.8%), Indonesia (12.8%), México (12%), and Peru (7%), among others. Considering the characteristics of the population, the mean age of the individuals was 42.5 years (15.1), and 255 (61.4%) were male. According to the resistant patterns, 223 strains (55.9%) were sensitive, and 176 (44.1%) were mono- poly-, multi-, pre-, and extremely DR. The most common resistance observed was to isoniazid in 35% (139 isolates), followed by rifampicin in 32% (127 isolates), and ethambutol in 21% (83 isolates).

According to the occurrence of T2DM, 175 genomes were from individuals with T2DM, of which 100 (57%) were drug-sensitive TB and 75 (43%) were DR-TB. The remaining 224 genomes belonged to patients without T2DM, of which 123 (55%) were drug-sensitive TB and 101 (45%) showed DR-TB. 

### 3.2. Sublineages Identified

All genomes were confirmed as belonging to L4, also known as Euro-American, and were distributed among seven sublineages. The sublineage 4.10, known as T and globally described, was observed in 101 (25%) isolates. Sublineage 4.6.1 (Cameron) was found in one isolate (0.2%), and sublineage 4.5 (H3,H4,T1) was observed in three (0.7%) strains. 

Two branches of sublineage 4.4 (S,T1,T2) were observed in thirteen isolates: 4.4.1.1 (S type) with ten strains (2.5%) and 4.4.1.2 (T1) with three (0.7%). 

Sublineage 4,3, also termed LAM, and widely distributed in Europe and Latin America, was identified in 111 isolates and distributed in five branches: 4.3.1 with 14 (3.5%) strains, 4.3.2 with 9 (2.2%), 4.3.3 with 71 (18%), 4.3.4.1 with 6 (1.4%), and 4.3.4.2 with 11 (2.7%) strains.

Sublineage 4.2 was observed in 48 strains and presented in two branches: 4.2.1 (H3,H4) with 47 (12%) strains and 4.2.2 (Lam7-TUR,T1) with one isolate. Sublineages 4.1, described as Harlem and widely distributed in Europe and Latin America, was observed in 109 isolates and included in four branches: 4.1.1 (X-family) with 7 (1.7%) isolates, 4.1.2 (T1,H1) with 8 (2%), 4.1.2.1 (T1,H1) with 81 (20%), and 4.1.1.3 (X3,X1) with 13 (3.2%) isolates.

### 3.3. Characterization of Variants and SNPs in the EPGs 

A total of 994 non-synonymous SNPs were identified in the 173 efflux pump genes analyzed. No variants were observed in 10 efflux pump genes (Rv0820, Rv1238, Rv1858, Rv2060, Rv2399c, Rv2937, Rv3501c, Rv3665c, Rv3666c, and Rv3756c), while 9 efflux pump genes showed between 1 and 3 variants (Rv0168, Rv0934, Rv0936, Rv1281c, Rv1680, Rv1964, Rv1965, Rv2832c, and Rv2835c), and 154 efflux pump genes showed between 3 and 19 variants (Supplementary Table S1). Table 1 shows the loci/genes with the highest number of changes, as well as the position and change in the most frequent of these. The loci Rv0194 and Rv2059 showed the highest number of variants, with 15 and 14, respectively. The most frequent non-synonymous mutations were S217P (position 1273071) at Rv1145 (*mmpL13a*) observed in 98 isolates, and M544I (position 1648620) at Rv1461, recorded in 78 isolates.

### 3.4. Characterization of Variants and SNPs in Efflux Pump Genes Associated with Sublineages

Table 2 shows the 29 changes found exclusively in the sublineages identified. Sublineage 4.2.1 showed seven mutations at six loci/genes, while 4.4.1 and 4.4.2 had six changes at five loci/genes, and 4.3.4.1 and 4.3.4.2 presented five changes at five loci/genes. Given the strong relationship between these variants and the sublineages, no further consideration of these was made in the subsequent comparative analysis, in order to avoid any bias. 

### 3.5. Efflux Pump Genes Variants in TB Genomes from Individuals with TB Drug Resistance and T2DM

Table 3 shows the 19 changes with the highest frequency of occurrence observed most frequently in isolates with DR, and the eight changes observed in eight genes found mainly in sensitive isolates, both of statistical significance. Six changes were observed in more than 20% of the resistant isolates, N105K (Rv0073), R189Q (Rv0173, *lprK*), V207L (Rv0411c, glnH), V77A (Rv0450c, *mmpL4*), Q334E (Rv1967, *mce3B*), and P161S (Rv1966, mce3A), while the change S217P (Rv1145) was reported in 29% of the sensitive drug isolates.

Table 4 shows the 40 changes of statistical significance present in the efflux pump genes in individuals with TB and T2DM. The frequencies of changes observed in individuals with T2DM were mainly low, with the most abundant being R140C (Rv3041c) found in 6% of isolates and M787V (Rv2339, narK1) in 5%. In TB patients, four changes presented frequencies higher than 19%: S217P (Rv1145, *mmpL13a*) in 32% of isolates, A291V (Rv1557, mmpL6) in 23.7%, P161S (Rv1966, mce3A) in 19%, and S220P (Rv2994) in 23.7%.

## 4. Discussion

Several reports describe a modification in the expression of efflux pump genes in the presence of certain drugs and the occurrence of specific variants associated with first- and second-line TB-DR in tuberculosis [12,25,26,27,28,29]. However, to our knowledge, this is the first report to characterize the variants in all of the efflux pump genes described to date in Mtb and to relate these in the context of possible association with DR and T2DM. The results evidence the occurrence of variants specifically associated with L4 sublineages and with the conditions TB-DR and T2DM.

Eleven genes showed the highest frequency of variants. The Rv1145 gene (*mmpL13a*) included the polymorphism S217P observed in 98 (25%) of the genomes analyzed. This gene has also been described as being overexpressed in the presence of anti-TB drugs, contributing to the overall resistance phenotype [26], and participating in the mechanism of resistance to pyrazinamide [30], even though this variant was observed mainly in sensitive isolates (Table 4). The efflux pumps Rv1967 (*mce3B*) and Rv0173 (*lprK*) also included mutations with a high frequency of occurrence. These genes are members of the Mce family and present a strong association mainly with virulence in TB [31]. The mutation S220P in Rv2994 was recorded in 75 (19%) strains. This gene encoded a transporter of the MFS family, which has been found to be highly expressed in MDR-TB isolates [32]. On the other hand, Rv0194 has been associated with the development of resistance against rifampicin [27,33]. Other variants found in efflux pump genes at high frequencies were M544I in the Rv1461 gene, found in 78 strains; variant A291V in Rv1557 (*mmpL6*), found in 75 strains; and variant R745Q in Rv0202c (*mmpL11*), observed in 57 isolates. Nevertheless, despite the high frequencies observed in our dataset, no function or association with DR has been described previously in these genes. These data show the great diversity of functions related to the genes carrying a significant number of variants, which could exert an influence at different levels of development in Mtb. 

In contrast, 19 efflux pump genes showed less than three SNPs or no changes at all. All of these genes belong to the ABC transporter family and are involved in the transport of different molecules, such as (i) inorganic ions (Rv0936 (pstA2), Rv1858 (*modB*), Rv2060 (Znub), Rv3666c (*dppA*), and Rv2399c (*cyst*)); (ii) organic molecules (Rv0820 (*PhoT*), Rv0934 (*pstS1*), Rv1281c (*oppD*), Rv1238 (*sugC*), Rv2832c (*ugpC*), Rv2835c (*ugpA*), Rv2937 (*drrB*), Rv3665c (*DppB*), and Rv3756c (*proZ*) (PZA)); and (iii) participating as integral membrane proteins (Rv3501c (*MlaE*), Rv0168 (*yrbE1B*), Rv1964 (*yrbE3A*), and Rv1965 (*yrbE3B*)). Only Rv3756c (*proZ*) was previously reported as being associated with resistance against pyrazinamide [28]. The low frequency or absence of variants in these genes suggests the importance of these pumps in the maintenance of the mycobacterial membrane function, so they could be considered as target for the design of new molecules to block their function, opening a whole new line of anti-tuberculosis adjunctive therapies.

To the best of our knowledge, this is the first description of 29 non-synonymous SNPs in efflux pump genes strongly related to 12 sublineages of L4 in Mtb. While the limited number of genomes analyzed here is acknowledged, along with the low representativeness of some sublineages, the clustering found, using those SNPs and the sublineages detected, raises new questions regarding the co-evolution of these mutations and the biological significance of these associations. It will undoubtedly be necessary to increase the number of isolates to determine the degree of conservation and coadaptation of these polymorphisms with sublineages, as well as their participation in the biology of mycobacteria.

Among the 19 genes that presented polymorphisms strongly associated with drug-resistant isolates, three families were observed: (i) the ABC family, which included five genes (Rv0073, Rv2038c, Rv3758c (*proV*), Rv0411c (*glnH*) and Rv0587, of which only Rv2038c has been described as related to MDR-TB [34]); (ii) the Mce family, which included Rv0171, (*mce1C*), Rv0173 (*lprK*), Rv1966 (*mce3A*), and Rv1967 (*mce3B*), all of which are associated mainly with virulence [31]; (iii) the Mycobacterial membrane proteins large family (*MmpL*), in which five genes were found (Rv0206c (*mmpL3*), Rv0450c (*mmpL4*), Rv0676c (*mmpL5*), Rv1522c (*mmpL12*), and Rv2942 (*mmpL7*)). Of these latter genes, Rv0450c and Rv2942 have been described as associated with DR [29,35], and Rv0676c has recently been related to resistance against bedaquiline [36]. Identification of these variants in DR isolates raises the need for further testing and developing additional assays to confirm their involvement in drug resistance.

The 19 polymorphic genes with the highest frequency in the TB-T2DM group were placed in three families: (1) the MmpLs, which have been described as necessary for the growth, resistance, nodulation, division, and survival of Mtb. These genes were Rv2339 (*narK1*), Rv0206c (*mmpL3*), Rv1146 (*mmpL13b*), Rv2942 (*mmpL7*), and Rv3823c (*mmpL8*), of which Rv1146 and Rv3823c have been found overexpressed in MDR-TB isolates [26] and those with high levels of resistance to isoniazid [37]. In addition, Rv0206 (*MmpL3*) is the only MmpL considered essential for the replication and viability of mycobacterial cells; (2) the ABC transporter family, comprising Rv0265c, Rv2041c, Rv3041c, Rv0986, Rv1273c, and Rv0587 (*yrbE2A*), of which only Rv1273c has been described as overexpressed in isoniazid-resistant isolates [37]; (3) the Mce family, Rv0170 (*mce1B*) and Rv0171 (*mce1C*), which has mainly been associated with Mtb virulence [27]. Finally, of the remaining six genes of no clear family (Rv0783c (*emrB*), Rv1146 (*mmpL13b*), Rv1619, Rv1667c, Rv1843c (*guaB1*), and Rv2265), only Rv1667c has been described as overexpressed in pyrazinamide-resistant isolates [28]. This extensive number of variant and pump families, with multiple functions, could be the result of the response of the bacteria to altered metabolic conditions in the host presenting T2DM. However, further studies are necessary to fully explore this possibility, including the potential effect of compensatory mutations. 

The major limitations of this study were as follows; Firstly, the reduced number of genomes and thus of the individuals included in this study generated an unmatched sample distribution in some of the groups. This was due to the absence of information in the metadata associated with the genomes and related to the clinical and epidemiological information of the hosts, including the occurrence of comorbidities such as T2DM, pharmacological treatment for glycemia control, glycemia tests, time elapsed with the T2DM condition, or substance addictions, among others; in this context, metadata is becoming a fundamental variable to guide the development of research, where it is intended to find associations between genomic variants and clinical variables, so it should be considered as an important element for the deposition of genomes in the repositories. The second limitation was the absence of information related to the phenotypic determination against all the drug resistances in the isolates whose genomes were included in this study; this information could address with more clarity the occurrence of some of the identified variants; however, this is difficult to obtain considering the diverse contexts in which these isolates were recovered and analyzed experimentally. The last limitation was that non-coding DNA associated with genetic regulation of efflux pump genes was not included in the analysis, and an important number of reports are associated with variants in these regions that modify the expression of these pumps. An additional limitation is related to the restriction of lineage 4, limiting the generalization of the results to other lineages such as lineage 1, widely recognized as a lineage with a significant tendency to develop drug resistance, and mainly distributed in countries from China and India, also important contributors of T2DM. 

## 5. Conclusions

The results show that the occurrence of 19 variants in EPG are apparently associated with DR, while 19 are possibly influenced by the presence of T2DM. These data support the possibility that the environment of a patient with T2DM could influence the generation of polymorphisms in *M. tuberculosis*. However, it is necessary to increase the number of genomes including other lineages and, importantly, to develop experimental studies to confirm the influence of the variants here identified to confirm their real participation and the influence of the DM2 in the induction of variants in *M. tuberculosis* in efflux pumps. 

## Figures and Tables

**Table 1:** EPGs with a high frequency of variants and SNPs in *M. tuberculosis*.

Locus (Gene)	Number of Genomes with Changes	No. of Changes	Position	Change	Frequency
Rv1145 (*mmpL13a*)	101	4	1273071	S217P	98
Rv1461	84	9	1648620	M544I	78
Rv2994	97	5	3351926	S220P	75
Rv1557 (*mmpL6*)	78	4	1762615	A291V	75
Rv2059	75	14	2315748	D192A	57
Rv0202c (*mmpL11*)	72	13	239059	R745Q	57
Rv1967 (*mce3B*)	82	11	2211600	Q334E	49
Rv0173 (*lprK*)	56	7	204630	R189Q	39
Rv0194	53	15	227286	V137M	25
Rv0450c (*mmpL4*)	53	11	541262	V77A	39

**Table 2:** SNPs in EPGs restricted to sublineages.

Sublineage	Locus (Gene)	Position	Change	Frequency
4.1.1.1-3	Rv2994	3352244	T326A	13
	Rv0783c (*emrB*)	877224	G406V	11
	Rv0987	1102788	V83F	11
	Rv0507 (*mmpL2*)	599165	E656A	15
4.2.1	Rv1819c (*bacA*)	2063911	I273T	47
	Rv0172	202675	I67T	47
	Rv3783 (*rfbD*)	4230033	V259A	47
	Rv2059	2316510	G446D	47
	Rv2059	2315669	V166I	47
	Rv3498c	3917305	V232F	47
	Rv3044 (*fecB*)	3406045	A304T	47
4.3.1	Rv0592 (*mce2D*)	691309	S270N	12
4.3.2	Rv2398c (*cysW*)	2695094	A236P	9
4.3.3	Rv1668c	1895174	D57N	71
4.3.4.1-2	Rv2326c	2599821	A43S	17
	Rv0933 (*pstB*)	1041445	T61M	17
	Rv1877	2126366	V155L	17
	Rv1672	197047	M63V	6
	Rv1967 (*mce3B*)	2210740	N47T	6
4.3.4.2	Rv0037c	40162	M347I	11
4.4.1.1-2	Rv0987	1105102	E854A	13
	Rv0987	1103786	L415F	13
	Rv1522c (*mmpL12*)	1715531	S694R	10
	Rv1619	1819488	R305Q	13
	Rv1968 (*mce3C*)	2211714	Y30C	10
	Rv0402c (*mmpL1*)	482418	G272R	10
4.10	Rv0930 (*pstA1*)	1037012	T5M	101
	Rv2688c	3005185	T156P	101
	Rv2398c (*cysW*)	2695378	A141G	101

**Table 3:** EPG SNPs in sensitive and resistant *M. tuberculosis* genomes from individuals with TB and TB-T2DM.

Locus (Gene)	Position	Change	Sensitive		Resistant		*p*-Value
			Frequency (N = 223)	%	Frequency (N = 176)	%	
***Resistant isolates***							
Rv0073	81990	N105K	4	1.8	35	20.0	0.000
Rv0171 (*mce1C*)	202279	P450T	0	0	9	5.1	0.001
Rv0173 (*lprK*)	204489	L142S	0	0	10	5.7	0.000
Rv0173 (*lprK*)	204630	R189Q	4	1.8	35	20.0	0.000
Rv0206c (*mmpL3*)	245080	G747R	0	0	9	5.1	0.001
Rv0411c (*glnH*)	497682	V207L	4	1.8	35	20.0	0.000
Rv0435c	524080	A152P	0	0	10	5.7	0.000
Rv0450c (*mmpL4*)	541262	V77A	4	1.8	35	20.0	0.000
Rv0587	685336	V70L	0	0	10	5.7	0.000
Rv0676c (*mmpL5*)	778107	S125C	0	0	5	2.8	0.016
Rv0783c (*emrB*)	877011	R477Q	0	0	9	5.1	0.001
Rv1522c (*mmpL12*)	1715219	E798D	0	0	5	2.8	0.016
Rv1619	1819507	E311D	0	0	9	5.1	0.001
Rv1667c	1893672	G187R	0	0	9	5.1	0.001
Rv1966 (*mce3A*)	2209807	P161S	12	5.4	38	21.6	0.000
Rv1967 (*mce3B*)	2211600	Q334E	12	5.4	37	21	0.000
Rv2038c	2284636	L54W	0	0	30	17	0.000
Rv2942 (*mmpL7*)	3287806	L913F	0	0	10	5.7	0.000
Rv3758c (*proV*)	4203468	V317A	0	0	5	2.8	0.016
***Sensitive isolates***							
Rv0194	227133	H86Y	7	3.1	0	0	0.019
Rv0194	227286	V137M	15	6.7	0	0	0.000
Rv0933 (*pstB*)	1042067	D268E	15	6.7	0	0	0.000
Rv1145	1273071	S217P	64	29	34	19	0.035
Rv1971 (*mce3F*)	2216394	T380P	15	6.7	0	0	0.000
Rv2329c (*narK1*)	2602760	M234I	7	3.1	0	0	0.019
Rv3041c	3401501	R140C	11	4.9	1	0.6	0.015
Rv3783 (*rfbD*)	4229625	V123A	14	6.3	0	0	0.000

**Table 4:** Efflux pump genes variants with significant differences in hosts with TB and TB-T2DM.

Locus (Gene)	Position	Change	Host with TB		Host with TB-T2DM		° *p*-Value
			N = 224	%	(N = 175)	%	
***TB-T2DM dominance***							
Rv0170 (*mce1B*)	200154	R85C	1	0.4	6	3.4	0.047
Rv0171 (*mce1C*)	202279	P450T *	2	0.9	7	4	0.046
Rv0206c (*mmpL3*)	244911	P803R	0	0	8	4.6	0.001
Rv0206c (*mmpL3*)	245080	G747R *	2	0.9	7	4	0.046
Rv0265c	317392	G38S	0	0	4	2.3	0.036
Rv0587 (*yrbE2A*)	685336	V70L *	2	0.9	8	4.6	0.025
Rv0783c (*emrB*)	877011	R477Q *	2	0.9	7	4	0.046
Rv0986	1101898	D32E	0	0	5	2.9	0.016
Rv1146 (*mmpL13b*)	1274703	L450S	1	0.4	6	3.4	0.047
Rv1273c	1423383	A223D	0	0	5	2.9	0.016
Rv1619	1819507	E311D *	2	0.9	7	4	0.046
Rv1667c	1893672	G187R *	2	0.9	7	4	0.038
Rv1843c (*guaB1*)	2093455	A82T	0	0	5	2.9	0.016
Rv2041c	2286715	R378G	0	0	5	2.9	0.016
Rv2265	2539687	V330I	0	0	6	3.4	0.007
Rv2339 (*narK1*)	2617051	M787V	0	0	9	5.1	0.001
Rv2942 (*mmpL7*)	3287806	L913F *	2	0.9	8	4.6	0.025
Rv3041c	3401501	R140C #	2	0.9	10	5.7	0.007
Rv3823c (*mmpL8*)	4291418	L38V	1	0.4	6	3.4	0.047
***TB dominance***							
Rv0073	81990	N105K *	32	14.3	7	4	0.001
Rv0173 (*lprK*) **	204630	R189Q *	32	14.3	7	4	0.001
Rv0194 **	227286	V137M #	15	6.7	0	0	0.000
Rv0202c (*mmpL11*) **	239059	R745Q	47	21	10	5.7	0.000
Rv0402c (*mmpL1*)	481374	R620G	21	9.4	6	3.4	0.026
Rv0411c (*glnH*)	497682	V207L *	32	14.3	7	4	0.001
Rv0450c (*mmpL4*) **	541262	V77A *	32	14.3	7	4	0.001
Rv0529 (*ccsA*)	619969	V27I	24	10.7	6	3.4	0.007
Rv0676c (*mmpL5*)	777451	V344L	8	3.6	0	0	0.011
Rv0933 (*pstB*)	1042067	D268E #	15	6.7	0	0	0.000
Rv1145 (*mmpL13a*) **	1273071	S217P #	72	32.1	26	14.9	0.000
Rv1218c	1361802	P311Q	8	3.6	0	0	0.011
Rv1557 (*mmpL6*) **	1762615	A291V	53	23.7	22	12.6	0.006
Rv1859	2107663	H364Y	9	4	0	0	0.006
Rv1966 (*mce3A*)	2209807	P161S	43	19.2	7	4	0.000
Rv1967 (*mce3*)	2211121	T174M	8	3.6	0	0	0.011
Rv1967 (*mce3B*) **	2211600	Q334E	42	18.8	7	4	0.000
Rv1971 (*mce3F*)	2216394	T380P #	15	6.7	0	0	0.000
Rv2038c	2284636	L54W	24	10.7	6	3.4	0.007
Rv2994 **	3351926	S220P	53	23.7	22	12.6	0.005
Rv3783 (*rfbD*)	4229625	V123A #	14	6.3	0	0	0.000

** Identified as high frequency variant. * Also identified as a variant related with DR isolates. # Also identified as a variant related with sensitive isolates. ° Fisher exact test (*p* ≤ 0.05).

## Data Availability

Code information of TB genomes used in this study are listed in Supplementary Materials, and can be freely accessed using the code assigned by the respective genome database.

## Supplementary Materials

The following supporting information can be downloaded at: https://www.mdpi.com/article/10.3390/microorganisms13061306/s1. Table S1: Information of the TB genomes used in this study.

## References

[1] Global Tuberculosis Report 2021. https://iris.who.int/bitstream/handle/10665/346387/9789240037021-eng.pdf?sequence=1.

[2] Restrepo B.I. (2016). Diabetes and tuberculosis. Microbiol. Spectr..

[3] Jeon C.Y., Murray M.B. (2008). Diabetes Mellitus Increases the Risk of Active Tuberculosis: A Systematic Review of 13 Observational Studies. PLoS Med..

[4] Pérez L.M., Restrepo B.I., Fuentes-Domínguez F.J., Duggirala R., Morales-Romero J., López-Alvarenga J.C., Comas I., Zenteno-Cuevas R. (2017). The effect size of type 2 diabetes mellitus on tuberculosis drug resistance and adverse treatment outcomes. Tuberculosis.

[5] Restrepo B.I., Schlesinger L.S. (2014). Impact of diabetes on the natural history of tuberculosis. Diabetes Res. Clin. Prac..

[6] Pérez-Navarro L.M., Fuentes-Domínguez F.J., Zenteno-Cuevas R. (2015). Type 2 diabetes mellitus and its influence in the development of multidrug resistance tuberculosis in patients from southeastern Mexico. J. Diabetes Complicat..

[7] Cornejo-Báez A.A., Zenteno-Cuevas R., Luna-Herrera J. (2024). Association Between Diabetes Mellitus-Tuberculosis and the Generation of Drug Resistance. Microorganisms.

[8] Dasan B., Rajamanickam A., Pandiarajan A.N., Shanmugam S., Nott S., Babu S. (2024). Immunological mechanisms of tuberculosis susceptibility in TB-infected individuals with type 2 diabetes mellitus: Insights from mycobacterial growth inhibition assay and cytokine analysis. Microbiol. Spectr..

[9] Sander P., Papavinasasundaram K.G., Dick T., Stavropoulos E., Ellrott K., Springer B., Colston M.J., Bo E.C. (2001). *Mycobacterium bovis* BCG recA deletion mutant shows increased susceptibility to DNA-damaging agents but wild-type survival in a mouse infection model. Infect. Immun..

[10] Singh A. (2017). Guardians of the mycobacterial genome: A review on DNA repair systems in *Mycobacterium tuberculosis*. Microbiology.

[11] Laws M., Jin P., Rahman K.M. (2021). Efflux pumps in *Mycobacterium tuberculosis* and their inhibition to tackle antimicrobial resistance. Trends Microbiol..

[12] Wang K., Pei H., Huang B., Zhu X., Zhang J., Zhou B., Zhu L., Zhang Y., Zhou F.-F. (2013). The expression of ABC efflux pump, Rv1217c-Rv1218c, and its association with multidrug resistance of *Mycobacterium tuberculosis* in China. Curr. Microbiol..

[13] Rodrigues L., Machado D., Couto I., Amaral L., Viveiros M. (2012). Contribution of efflux activity to isoniazid resistance in the *Mycobacterium tuberculosis* complex. Infect. Genet. Evol..

[14] Pérez-Lago L., Comas I., Navarro Y., González-Candelas F., Herranz M., Bouza E., García-de-Viedma D. (2014). Whole Genome Sequencing Analysis of Intrapatient Microevolution in *Mycobacterium tuberculosis*: Potential Impact on the Inference of Tuberculosis Transmission. J. Infect. Dis..

[15] Nimmo C., Brien K., Millard J., Grant A.D., Padayatchi N., Pym A.S., Max O’Donnell, Goldstein R., Breuer J., Balloux F. (2020). Dynamics of within-host *Mycobacterium tuberculosis* diversity and heteroresistance during treatment. EBioMedicine.

[16] Liu Q., Wei J., Li Y., Wang M., Su J., Lu Y., López M.G., Qian X., Zhu Z., Wang H. (2020). *Mycobacterium tuberculosis* clinical isolates carry mutational signatures of host immune environments. Sci. Adv..

[17] Bermudez-Hernández G.A., Pérez-Martínez D.E., Madrazo-Moya C.F., Cancino-Muñoz I., Comas I., Zenteno-Cuevas R. (2022). Whole genome sequencing analysis to evaluate the influence of T2DM on polymorphisms associated with drug resistance in M. tuberculosis. BMC Genom..

[18] Pérez-Martínez D.E., Bermúdez-Hernández G.A., Madrazo-Moya C.F., Cancino-Muñoz I., Montero H., Licona-Cassani C., Muñiz-Salazar R., Comas I., Zenteno-Cuevas R. (2022). SNPs in Genes Related to DNA Damage Repair in Mycobacterium Tuberculosis: Their Association with Type 2 Diabetes Mellitus and Drug Resistance. Genes.

[19] Picard Tools—By Broad Institute. https://broadinstitute.github.io/picard/.

[20] Chen S., Zhou Y., Chen Y., Gu J. (2018). fastp: An ultra-fast all-in-one FASTQ preprocessor. Bioinformatics.

[21] Wood D.E., Salzberg S.L. (2014). Kraken: Ultrafast metagenomic sequence classification using exact alignments. Genome Biol..

[22] Comas I., Coscolla M., Luo T., Borrell S., Holt K.E., Kato-Maeda M., Parkhill J., Malla B., Berg S., Thwaites G. (2013). Out-of-Africa migration and Neolithic coexpansion of *Mycobacterium tuberculosis* with modern humans. Nat. Genet..

[23] Coll F., McNerney R., Guerra-Assunção J.A., Glynn J.R., Perdigão J., Viveiros M., Portugal I., Pain A., Martin N. (2014). Clark A robust SNP barcode for typing *Mycobacterium tuberculosis* complex strains. Nat. Commun..

[24] IBM Corp (2020). IBM SPSS Statistics for Windows 2020, version 27.

[25] Rodrigues L., Baptista P., Veigas B., Amaral L., Viveiros M. (2012). Contribution of Efflux to the Emergence of Isoniazid and Multidrug Resistance in *Mycobacterium tuberculosis*. PLoS ONE.

[26] Ghajavand H., Kargarpour Kamakoli M., Khanipour S., Pourazar Dizaji S., Masoumi M., Rahimi Jamnani F., Fateh A., Yaseri M., Siadat S.D. (2019). Scrutinizing the drug resistance mechanism of multi- and extensively-drug resistant *Mycobacterium tuberculosis*: Mutations versus efflux pumps. Antimicrob. Resist. Infect. Control.

[27] Narang A., Garima K., Porwal S., Bhandekar A., Shrivastava K., Giri A., Sharma N.K., Bose M., Varma-Basil M. (2019). Potential impact of efflux pump genes in mediating rifampicin resistance in clinical isolates of *Mycobacterium tuberculosis* from India. PLoS ONE.

[28] Zhang Y., Zhang J., Cui P., Zhang Y., Zhang W. (2017). Identification of Novel Efflux Proteins Rv0191, Rv3756c, Rv3008, and Rv1667c Involved in Pyrazinamide Resistance in *Mycobacterium tuberculosis*. Antimicrob. Agents Chemother..

[29] Shahi F., Khosravi A.D., Tabandeh M.R., Salmanzadeh S. (2021). Investigation of the Rv3065, Rv2942, Rv1258c, Rv1410c, and Rv2459 efflux pump genes expression among multidrug-resistant *Mycobacterium tuberculosis* clinical isolates. Heliyon.

[30] Wang X., Ding B., Li B. (2013). Biomimetic electrospun nanofibrous structures for tissue engineering. Mater. Today.

[31] Klepp L.I., y Garcia J.S., FabianaBigi (2022). Mycobacterial MCE proteins as transporters that control lipid homeostasis of the cell wall. Tuberculosis.

[32] Li G., Zhang J., Guo Q., Jiang Y., Wei J., Zhao L.L., Zhao X., Lu J., Wan K. (2015). Efflux pump gene expression in multidrug-resistant *Mycobacterium tuberculosis* clinical isolates. PLoS ONE.

[33] Kanji A., Hasan R., Ali A., Zaver A., Zhang Y., Imtiaz K., Shi W., Clark T.G., McNerney R., Phelan J. (2017). Single nucleotide polymorphisms in efflux pumps genes in extensively drug resistant *Mycobacterium tuberculosis* isolates from Pakistan. Tuberculosis.

[34] Lee J.J., Kang H.Y., Lee W.I., Cho S.Y., Kim Y.J., Lee H.J. (2021). Efflux pump gene expression study using RNA-seq in multidrug-resistant TB. Int. J. Tuberc. Lung Dis..

[35] De Keijzer J., De Haas P.E., De Ru A.H., Van Veelen P.A., Van Soolingen D. (2014). Disclosure of selective advantages in the “modern” sublineage of the *Mycobacterium tuberculosis* Beijing genotype family by quantitative proteomics. Mol. Cell Proteom..

[36] Rivière E., Verboven L., Dippenaar A., Goossens S., De Vos E., Streicher E., Cuypers B., Laukens K., Ben-Rached F., Rodwell T.C. (2022). Variants in Bedaquiline-Candidate-Resistance Genes: Prevalence in Bedaquiline-Naive Patients, Effect on MIC, and Association with *Mycobacterium tuberculosis* Lineage. Antimicrob Agents Chemother.

[37] Narang A., Giri A., Gupta S., Garima K., Bose M., Varma-Basil M. (2017). Contribution of putative efflux pump genes to isoniazid resistance in clinical isolates of *Mycobacterium tuberculosis*. Int. J. Mycobacteriology.

