# Supplementary material for: Whole-Genome Sequence Analysis to Assess Mutations in Efflux Pumps in Mycobacterium tuberculosis: The Influence in Drug Resistance
Source: Microorganisms. 2025 Jun 4;13(6):1306. doi: 10.3390/microorganisms13061306 (PMC12195134; doi:10.3390/microorganisms13061306)
Supplement: Supplementary file 1 [file microorganisms-13-01306-s001.zip › microorganisms-3474243-supplementary.pdf]

Table S1. Information of the TB genomes used in this study

Information of the TB genomes used in this study				
ID	Country	Type 2 Diabetes mellitus	Drug resistance condition	Source
SRR5153073	Azerbaijan	YES	sensitive	Available at TB portals https://tbportals.niaid.nih.gov/
SRR6356936	Azerbaijan	YES	resistant	Available at TB portals https://tbportals.niaid.nih.gov/
SRR6356938	Azerbaijan	YES	resistant	Available at TB portals https://tbportals.niaid.nih.gov/
SRR6356975	Azerbaijan	YES	sensitive	Available at TB portals https://tbportals.niaid.nih.gov/
SRR6357008	Azerbaijan	YES	resistant	Available at TB portals https://tbportals.niaid.nih.gov/
SRR13232428	Belarus	No	resistant	Available at TB portals https://tbportals.niaid.nih.gov/
SRR13232467	Belarus	YES	resistant	Available at TB portals https://tbportals.niaid.nih.gov/
SRR13232498	Belarus	No	resistant	Available at TB portals https://tbportals.niaid.nih.gov/
SRR13232543	Belarus	No	resistant	Available at TB portals https://tbportals.niaid.nih.gov/
SRR13232554	Belarus	No	resistant	Available at TB portals https://tbportals.niaid.nih.gov/
SRR13232573	Belarus	YES	resistant	Available at TB portals https://tbportals.niaid.nih.gov/
SRR13232580	Belarus	No	resistant	Available at TB portals https://tbportals.niaid.nih.gov/
SRR13232591	Belarus	YES	resistant	Available at TB portals https://tbportals.niaid.nih.gov/
SRR13232681	Belarus	No	resistant	Available at TB portals https://tbportals.niaid.nih.gov/
SRR13232706	Belarus	No	resistant	Available at TB portals https://tbportals.niaid.nih.gov/
SRR13232756	Belarus	No	resistant	Available at TB portals https://tbportals.niaid.nih.gov/
SRR13232779	Belarus	YES	resistant	Available at TB portals https://tbportals.niaid.nih.gov/
SRR6458398	Belarus	No	sensitive	Available at TB portals https://tbportals.niaid.nih.gov/
SRR6458409	Belarus	YES	sensitive	Available at TB portals https://tbportals.niaid.nih.gov/
SRR6458433	Belarus	No	resistant	Available at TB portals https://tbportals.niaid.nih.gov/
SRR6458436	Belarus	No	resistant	Available at TB portals https://tbportals.niaid.nih.gov/
SRR6458461	Belarus	No	sensitive	Available at TB portals https://tbportals.niaid.nih.gov/
SRR6458462	Belarus	No	resistant	Available at TB portals https://tbportals.niaid.nih.gov/
SRR6458464	Belarus	No	sensitive	Available at TB portals https://tbportals.niaid.nih.gov/
SRR10397092	Georgia	No	resistant	Available at TB portals https://tbportals.niaid.nih.gov/
SRR10397107	Georgia	YES	resistant	Available at TB portals https://tbportals.niaid.nih.gov/
SRR10397108	Georgia	No	resistant	Available at TB portals https://tbportals.niaid.nih.gov/

SRR10397114	Georgia	No	resistant	Available at TB portals https://tbportals.niaid.nih.gov/
SRR10397170	Georgia	YES	resistant	Available at TB portals https://tbportals.niaid.nih.gov/
SRR10397186	Georgia	No	resistant	Available at TB portals https://tbportals.niaid.nih.gov/
SRR10397193	Georgia	No	resistant	Available at TB portals https://tbportals.niaid.nih.gov/
SRR10397202	Georgia	No	sensitive	Available at TB portals https://tbportals.niaid.nih.gov/
SRR10397218	Georgia	YES	resistant	Available at TB portals https://tbportals.niaid.nih.gov/
SRR10397250	Georgia	No	sensitive	Available at TB portals https://tbportals.niaid.nih.gov/
SRR10397253	Georgia	No	resistant	Available at TB portals https://tbportals.niaid.nih.gov/
SRR10397258	Georgia	No	sensitive	Available at TB portals https://tbportals.niaid.nih.gov/
SRR10397266	Georgia	No	resistant	Available at TB portals https://tbportals.niaid.nih.gov/
SRR10397269	Georgia	No	sensitive	Available at TB portals https://tbportals.niaid.nih.gov/
SRR11033589	Georgia	No	sensitive	Available at TB portals https://tbportals.niaid.nih.gov/
SRR11033590	Georgia	No	sensitive	Available at TB portals https://tbportals.niaid.nih.gov/
SRR11033593	Georgia	No	resistant	Available at TB portals https://tbportals.niaid.nih.gov/
SRR11033594	Georgia	No	sensitive	Available at TB portals https://tbportals.niaid.nih.gov/
SRR11033595	Georgia	No	sensitive	Available at TB portals https://tbportals.niaid.nih.gov/
SRR11033596	Georgia	No	sensitive	Available at TB portals https://tbportals.niaid.nih.gov/
SRR11033597	Georgia	No	sensitive	Available at TB portals https://tbportals.niaid.nih.gov/
SRR11033600	Georgia	No	resistant	Available at TB portals https://tbportals.niaid.nih.gov/
SRR11033601	Georgia	No	sensitive	Available at TB portals https://tbportals.niaid.nih.gov/
SRR11033602	Georgia	No	sensitive	Available at TB portals https://tbportals.niaid.nih.gov/
SRR11033604	Georgia	No	sensitive	Available at TB portals https://tbportals.niaid.nih.gov/
SRR11033605	Georgia	No	sensitive	Available at TB portals https://tbportals.niaid.nih.gov/
SRR11033606	Georgia	No	sensitive	Available at TB portals https://tbportals.niaid.nih.gov/
SRR11033607	Georgia	No	sensitive	Available at TB portals https://tbportals.niaid.nih.gov/
SRR11033608	Georgia	No	sensitive	Available at TB portals https://tbportals.niaid.nih.gov/
SRR11033609	Georgia	No	sensitive	Available at TB portals https://tbportals.niaid.nih.gov/
SRR11033611	Georgia	No	resistant	Available at TB portals https://tbportals.niaid.nih.gov/
SRR11033612	Georgia	No	sensitive	Available at TB portals https://tbportals.niaid.nih.gov/
SRR11033613	Georgia	No	sensitive	Available at TB portals https://tbportals.niaid.nih.gov/

SRR11033615	Georgia	No	sensitive	Available at TB portals https://tbportals.niaid.nih.gov/
SRR11033617	Georgia	No	sensitive	Available at TB portals https://tbportals.niaid.nih.gov/
SRR11033618	Georgia	No	sensitive	Available at TB portals https://tbportals.niaid.nih.gov/
SRR11033619	Georgia	No	sensitive	Available at TB portals https://tbportals.niaid.nih.gov/
SRR11033620	Georgia	No	resistant	Available at TB portals https://tbportals.niaid.nih.gov/
SRR11033622	Georgia	No	sensitive	Available at TB portals https://tbportals.niaid.nih.gov/
SRR11033623	Georgia	No	sensitive	Available at TB portals https://tbportals.niaid.nih.gov/
SRR11033624	Georgia	No	sensitive	Available at TB portals https://tbportals.niaid.nih.gov/
SRR11033625	Georgia	No	resistant	Available at TB portals https://tbportals.niaid.nih.gov/
SRR11033626	Georgia	No	sensitive	Available at TB portals https://tbportals.niaid.nih.gov/
SRR11033627	Georgia	No	sensitive	Available at TB portals https://tbportals.niaid.nih.gov/
SRR11033628	Georgia	No	sensitive	Available at TB portals https://tbportals.niaid.nih.gov/
SRR11033631	Georgia	No	sensitive	Available at TB portals https://tbportals.niaid.nih.gov/
SRR11033633	Georgia	No	sensitive	Available at TB portals https://tbportals.niaid.nih.gov/
SRR11033634	Georgia	No	resistant	Available at TB portals https://tbportals.niaid.nih.gov/
SRR11033635	Georgia	No	sensitive	Available at TB portals https://tbportals.niaid.nih.gov/
SRR11033637	Georgia	No	resistant	Available at TB portals https://tbportals.niaid.nih.gov/
SRR11033638	Georgia	No	sensitive	Available at TB portals https://tbportals.niaid.nih.gov/
SRR11033642	Georgia	No	sensitive	Available at TB portals https://tbportals.niaid.nih.gov/
SRR11033646	Georgia	No	resistant	Available at TB portals https://tbportals.niaid.nih.gov/
SRR11033647	Georgia	No	sensitive	Available at TB portals https://tbportals.niaid.nih.gov/
SRR11033648	Georgia	No	sensitive	Available at TB portals https://tbportals.niaid.nih.gov/
SRR11033649	Georgia	No	resistant	Available at TB portals https://tbportals.niaid.nih.gov/
SRR11033650	Georgia	No	sensitive	Available at TB portals https://tbportals.niaid.nih.gov/
SRR11033652	Georgia	No	sensitive	Available at TB portals https://tbportals.niaid.nih.gov/
SRR11033653	Georgia	No	sensitive	Available at TB portals https://tbportals.niaid.nih.gov/
SRR11033656	Georgia	No	sensitive	Available at TB portals https://tbportals.niaid.nih.gov/
SRR11033657	Georgia	No	sensitive	Available at TB portals https://tbportals.niaid.nih.gov/
SRR11033658	Georgia	No	resistant	Available at TB portals https://tbportals.niaid.nih.gov/
SRR11033659	Georgia	No	sensitive	Available at TB portals https://tbportals.niaid.nih.gov/

SRR11033660	Georgia	No	sensitive	Available at TB portals https://tbportals.niaid.nih.gov/
SRR11033666	Georgia	No	sensitive	Available at TB portals https://tbportals.niaid.nih.gov/
SRR11033667	Georgia	No	sensitive	Available at TB portals https://tbportals.niaid.nih.gov/
SRR11033669	Georgia	No	sensitive	Available at TB portals https://tbportals.niaid.nih.gov/
SRR11033670	Georgia	No	sensitive	Available at TB portals https://tbportals.niaid.nih.gov/
SRR11033672	Georgia	No	sensitive	Available at TB portals https://tbportals.niaid.nih.gov/
SRR11033681	Georgia	No	resistant	Available at TB portals https://tbportals.niaid.nih.gov/
SRR11033682	Georgia	No	sensitive	Available at TB portals https://tbportals.niaid.nih.gov/
SRR11033683	Georgia	No	sensitive	Available at TB portals https://tbportals.niaid.nih.gov/
SRR11033684	Georgia	No	resistant	Available at TB portals https://tbportals.niaid.nih.gov/
SRR11033687	Georgia	No	sensitive	Available at TB portals https://tbportals.niaid.nih.gov/
SRR11033689	Georgia	No	sensitive	Available at TB portals https://tbportals.niaid.nih.gov/
SRR11033697	Georgia	No	sensitive	Available at TB portals https://tbportals.niaid.nih.gov/
SRR11033700	Georgia	No	resistant	Available at TB portals https://tbportals.niaid.nih.gov/
SRR11033702	Georgia	No	resistant	Available at TB portals https://tbportals.niaid.nih.gov/
SRR11033707	Georgia	No	sensitive	Available at TB portals https://tbportals.niaid.nih.gov/
SRR11033708	Georgia	No	sensitive	Available at TB portals https://tbportals.niaid.nih.gov/
SRR11033709	Georgia	No	sensitive	Available at TB portals https://tbportals.niaid.nih.gov/
SRR11033712	Georgia	No	resistant	Available at TB portals https://tbportals.niaid.nih.gov/
SRR11033714	Georgia	No	resistant	Available at TB portals https://tbportals.niaid.nih.gov/
SRR11033720	Georgia	No	sensitive	Available at TB portals https://tbportals.niaid.nih.gov/
SRR11033723	Georgia	No	sensitive	Available at TB portals https://tbportals.niaid.nih.gov/
SRR11033725	Georgia	No	sensitive	Available at TB portals https://tbportals.niaid.nih.gov/
SRR11033733	Georgia	No	sensitive	Available at TB portals https://tbportals.niaid.nih.gov/
SRR11033749	Georgia	No	sensitive	Available at TB portals https://tbportals.niaid.nih.gov/
SRR11033754	Georgia	No	sensitive	Available at TB portals https://tbportals.niaid.nih.gov/
SRR11033757	Georgia	No	resistant	Available at TB portals https://tbportals.niaid.nih.gov/
SRR11033762	Georgia	No	resistant	Available at TB portals https://tbportals.niaid.nih.gov/
SRR11033763	Georgia	No	sensitive	Available at TB portals https://tbportals.niaid.nih.gov/
SRR11033764	Georgia	No	sensitive	Available at TB portals https://tbportals.niaid.nih.gov/

SRR11033765	Georgia	No	sensitive	Available at TB portals https://tbportals.niaid.nih.gov/
SRR11033766	Georgia	No	sensitive	Available at TB portals https://tbportals.niaid.nih.gov/
SRR11033769	Georgia	No	sensitive	Available at TB portals https://tbportals.niaid.nih.gov/
SRR11033770	Georgia	No	sensitive	Available at TB portals https://tbportals.niaid.nih.gov/
SRR11033771	Georgia	No	sensitive	Available at TB portals https://tbportals.niaid.nih.gov/
SRR11033773	Georgia	No	sensitive	Available at TB portals https://tbportals.niaid.nih.gov/
SRR11033774	Georgia	No	sensitive	Available at TB portals https://tbportals.niaid.nih.gov/
SRR11033776	Georgia	No	sensitive	Available at TB portals https://tbportals.niaid.nih.gov/
SRR11033777	Georgia	No	sensitive	Available at TB portals https://tbportals.niaid.nih.gov/
SRR11033780	Georgia	No	sensitive	Available at TB portals https://tbportals.niaid.nih.gov/
SRR3544717	Georgia	No	resistant	Available at TB portals https://tbportals.niaid.nih.gov/
SRR3544722	Georgia	No	sensitive	Available at TB portals https://tbportals.niaid.nih.gov/
SRR3544731	Georgia	No	resistant	Available at TB portals https://tbportals.niaid.nih.gov/
SRR3544736	Georgia	No	sensitive	Available at TB portals https://tbportals.niaid.nih.gov/
SRR3544738	Georgia	No	sensitive	Available at TB portals https://tbportals.niaid.nih.gov/
SRR3544742	Georgia	No	sensitive	Available at TB portals https://tbportals.niaid.nih.gov/
SRR3544744	Georgia	No	sensitive	Available at TB portals https://tbportals.niaid.nih.gov/
SRR3544750	Georgia	No	sensitive	Available at TB portals https://tbportals.niaid.nih.gov/
SRR3544752	Georgia	No	sensitive	Available at TB portals https://tbportals.niaid.nih.gov/
SRR5152915	Georgia	No	sensitive	Available at TB portals https://tbportals.niaid.nih.gov/
SRR5152927	Georgia	No	resistant	Available at TB portals https://tbportals.niaid.nih.gov/
SRR5152929	Georgia	No	sensitive	Available at TB portals https://tbportals.niaid.nih.gov/
SRR5152938	Georgia	No	sensitive	Available at TB portals https://tbportals.niaid.nih.gov/
SRR5153088	Georgia	YES	resistant	Available at TB portals https://tbportals.niaid.nih.gov/
SRR5153089	Georgia	No	resistant	Available at TB portals https://tbportals.niaid.nih.gov/
SRR5153095	Georgia	No	resistant	Available at TB portals https://tbportals.niaid.nih.gov/
SRR5153213	Georgia	No	sensitive	Available at TB portals https://tbportals.niaid.nih.gov/
SRR5153216	Georgia	No	resistant	Available at TB portals https://tbportals.niaid.nih.gov/
SRR5153240	Georgia	No	resistant	Available at TB portals https://tbportals.niaid.nih.gov/
SRR5153266	Georgia	No	sensitive	Available at TB portals https://tbportals.niaid.nih.gov/

SRR5153309	Georgia	No	resistant	Available at TB portals https://tbportals.niaid.nih.gov/
SRR5153316	Georgia	No	resistant	Available at TB portals https://tbportals.niaid.nih.gov/
SRR5153332	Georgia	YES	resistant	Available at TB portals https://tbportals.niaid.nih.gov/
SRR7516353	Georgia	YES	resistant	Available at TB portals https://tbportals.niaid.nih.gov/
SRR7516357	Georgia	No	sensitive	Available at TB portals https://tbportals.niaid.nih.gov/
SRR7516400	Georgia	No	resistant	Available at TB portals https://tbportals.niaid.nih.gov/
SRR7516407	Georgia	No	resistant	Available at TB portals https://tbportals.niaid.nih.gov/
SRR7516420	Georgia	No	sensitive	Available at TB portals https://tbportals.niaid.nih.gov/
SRR7516434	Georgia	No	resistant	Available at TB portals https://tbportals.niaid.nih.gov/
SRR7516437	Georgia	YES	resistant	Available at TB portals https://tbportals.niaid.nih.gov/
SRR7516445	Georgia	No	sensitive	Available at TB portals https://tbportals.niaid.nih.gov/
SRR10808355	Indonesia	YES	sensitive	Available at ENA portals https://www.ebi.ac.uk/ena/browser/home
SRR10808388	Indonesia	YES	sensitive	Available at ENA portals https://www.ebi.ac.uk/ena/browser/home
SRR10808432	Indonesia	YES	sensitive	Available at ENA portals https://www.ebi.ac.uk/ena/browser/home
SRR10808450	Indonesia	YES	sensitive	Available at ENA portals https://www.ebi.ac.uk/ena/browser/home
SRR10808546	Indonesia	YES	resistant	Available at ENA portals https://www.ebi.ac.uk/ena/browser/home
SRR10808568	Indonesia	YES	sensitive	Available at ENA portals https://www.ebi.ac.uk/ena/browser/home /
SRR10808708	Indonesia	YES	sensitive	Available at ENA portals https://www.ebi.ac.uk/ena/browser/home
SRR10808712	Indonesia	YES	sensitive	Available at ENA portals https://www.ebi.ac.uk/ena/browser/home /
SRR10808713	Indonesia	YES	sensitive	Available at ENA portals https://www.ebi.ac.uk/ena/browser/home
SRR10808722	Indonesia	YES	sensitive	Available at ENA portals https://www.ebi.ac.uk/ena/browser/home /
SRR10808725	Indonesia	YES	sensitive	Available at ENA portals https://www.ebi.ac.uk/ena/browser/home
SRR10808739	Indonesia	YES	sensitive	Available at ENA portals https://www.ebi.ac.uk/ena/browser/home /

SRR10808758	Indonesia	YES	sensitive	Available at ENA portals https://www.ebi.ac.uk/ena/browser/home
SRR10808770	Indonesia	YES	resistant	Available at ENA portals https://www.ebi.ac.uk/ena/browser/home /
SRR10808811	Indonesia	YES	sensitive	Available at ENA portals https://www.ebi.ac.uk/ena/browser/home
SRR10808934	Indonesia	YES	sensitive	Available at ENA portals https://www.ebi.ac.uk/ena/browser/home /
SRR10808965	Indonesia	YES	sensitive	Available at ENA portals https://www.ebi.ac.uk/ena/browser/home
SRR10808969	Indonesia	YES	sensitive	Available at ENA portals https://www.ebi.ac.uk/ena/browser/home /
SRR10808970	Indonesia	YES	resistant	Available at ENA portals https://www.ebi.ac.uk/ena/browser/home
SRR10808990	Indonesia	YES	sensitive	Available at ENA portals https://www.ebi.ac.uk/ena/browser/home /
SRR10808996	Indonesia	YES	resistant	Available at ENA portals https://www.ebi.ac.uk/ena/browser/home
SRR10809020	Indonesia	YES	sensitive	Available at ENA portals https://www.ebi.ac.uk/ena/browser/home /
SRR10809030	Indonesia	YES	sensitive	Available at ENA portals https://www.ebi.ac.uk/ena/browser/home
SRR10809047	Indonesia	YES	sensitive	Available at ENA portals https://www.ebi.ac.uk/ena/browser/home /
SRR10809049	Indonesia	YES	sensitive	Available at ENA portals https://www.ebi.ac.uk/ena/browser/home
SRR10809050	Indonesia	YES	sensitive	Available at ENA portals https://www.ebi.ac.uk/ena/browser/home /
SRR10809054	Indonesia	YES	sensitive	Available at ENA portals https://www.ebi.ac.uk/ena/browser/home /
SRR10809068	Indonesia	YES	sensitive	Available at ENA portals https://www.ebi.ac.uk/ena/browser/home
SRR10809070	Indonesia	YES	sensitive	Available at ENA portals https://www.ebi.ac.uk/ena/browser/home /
SRR10809072	Indonesia	YES	sensitive	Available at ENA portals https://www.ebi.ac.uk/ena/browser/home
SRR10809083	Indonesia	YES	sensitive	Available at ENA portals https://www.ebi.ac.uk/ena/browser/home /

SRR10809084	Indonesia	YES	sensitive	Available at ENA portals https://www.ebi.ac.uk/ena/browser/home
SRR10809085	Indonesia	YES	sensitive	Available at ENA portals https://www.ebi.ac.uk/ena/browser/home /
SRR10809086	Indonesia	YES	resistant	Available at ENA portals https://www.ebi.ac.uk/ena/browser/home
SRR10809089	Indonesia	YES	sensitive	Available at ENA portals https://www.ebi.ac.uk/ena/browser/home /
SRR10809103	Indonesia	YES	resistant	Available at ENA portals https://www.ebi.ac.uk/ena/browser/home /
SRR10809104	Indonesia	YES	sensitive	Available at ENA portals https://www.ebi.ac.uk/ena/browser/home
SRR10809113	Indonesia	YES	sensitive	Available at ENA portals https://www.ebi.ac.uk/ena/browser/home /
SRR10809116	Indonesia	YES	sensitive	Available at ENA portals https://www.ebi.ac.uk/ena/browser/home
SRR10809123	Indonesia	YES	resistant	Available at ENA portals https://www.ebi.ac.uk/ena/browser/home /
SRR10809136	Indonesia	YES	sensitive	Available at ENA portals https://www.ebi.ac.uk/ena/browser/home
SRR10809137	Indonesia	YES	sensitive	Available at ENA portals https://www.ebi.ac.uk/ena/browser/home /
SRR10809140	Indonesia	YES	resistant	Available at ENA portals https://www.ebi.ac.uk/ena/browser/home
SRR10809144	Indonesia	YES	sensitive	Available at ENA portals https://www.ebi.ac.uk/ena/browser/home /
SRR10809160	Indonesia	YES	sensitive	Available at ENA portals https://www.ebi.ac.uk/ena/browser/home /
SRR10809171	Indonesia	YES	sensitive	Available at ENA portals https://www.ebi.ac.uk/ena/browser/home
SRR10809189	Indonesia	YES	sensitive	Available at ENA portals https://www.ebi.ac.uk/ena/browser/home /
SRR10809194	Indonesia	YES	sensitive	Available at ENA portals https://www.ebi.ac.uk/ena/browser/home
SRR10809199	Indonesia	YES	sensitive	Available at ENA portals https://www.ebi.ac.uk/ena/browser/home /
SRR10809207	Indonesia	YES	resistant	Available at ENA portals https://www.ebi.ac.uk/ena/browser/home

SRR10809213	Indonesia	YES	sensitive	Available at ENA portals https://www.ebi.ac.uk/ena/browser/home /
SRR13232424	Kazakhstan	No	resistant	Available at TB portals https://tbportals.niaid.nih.gov/
SRR13232487	Kazakhstan	No	sensitive	Available at TB portals https://tbportals.niaid.nih.gov/
SRR13232622	Kazakhstan	YES	sensitive	Available at TB portals https://tbportals.niaid.nih.gov/
ERR3148156	Mexico	No	resistant	Available at ENA portals https://www.ebi.ac.uk/ena/browser/home /
ERR3148157	Mexico	YES	sensitive	Available at ENA portals https://www.ebi.ac.uk/ena/browser/home
ERR3148163	Mexico	YES	resistant	Available at ENA portals https://www.ebi.ac.uk/ena/browser/home /
ERR3148165	Mexico	YES	resistant	Available at ENA portals https://www.ebi.ac.uk/ena/browser/home
ERR3148170	Mexico	YES	resistant	Available at ENA portals https://www.ebi.ac.uk/ena/browser/home /
ERR3148171	Mexico	YES	resistant	Available at ENA portals https://www.ebi.ac.uk/ena/browser/home
ERR3148172	Mexico	YES	resistant	Available at ENA portals https://www.ebi.ac.uk/ena/browser/home /
ERR3148173	Mexico	YES	resistant	Available at ENA portals https://www.ebi.ac.uk/ena/browser/home
ERR3148174	Mexico	YES	resistant	Available at ENA portals https://www.ebi.ac.uk/ena/browser/home /
ERR3148175	Mexico	YES	resistant	Available at ENA portals https://www.ebi.ac.uk/ena/browser/home
ERR3148176	Mexico	YES	resistant	Available at ENA portals https://www.ebi.ac.uk/ena/browser/home /
ERR3148184	Mexico	No	resistant	Available at ENA portals https://www.ebi.ac.uk/ena/browser/home
ERR3148186	Mexico	YES	resistant	Available at ENA portals https://www.ebi.ac.uk/ena/browser/home /
ERR3148187	Mexico	YES	resistant	Available at TB portals https://tbportals.niaid.nih.gov/
ERR3148188	Mexico	YES	resistant	Available at ENA portals https://www.ebi.ac.uk/ena/browser/home /
ERR3148189	Mexico	YES	resistant	Available at ENA portals https://www.ebi.ac.uk/ena/browser/home
ERR3148190	Mexico	YES	resistant	Available at ENA portals https://www.ebi.ac.uk/ena/browser/home /

ERR3148191	Mexico	YES	resistant	Available at ENA portals https://www.ebi.ac.uk/ena/browser/home
ERR3148192	Mexico	YES	resistant	Available at ENA portals https://www.ebi.ac.uk/ena/browser/home /
ERR3148193	Mexico	No	sensitive	Available at ENA portals https://www.ebi.ac.uk/ena/browser/home
ERR3148195	Mexico	YES	sensitive	Available at ENA portals https://www.ebi.ac.uk/ena/browser/home /
ERR3148196	Mexico	YES	sensitive	Available at ENA portals https://www.ebi.ac.uk/ena/browser/home
ERR3148197	Mexico	YES	resistant	Available at ENA portals https://www.ebi.ac.uk/ena/browser/home /
ERR3148198	Mexico	No	resistant	Available at ENA portals https://www.ebi.ac.uk/ena/browser/home /
ERR3148199	Mexico	No	resistant	Available at ENA portals https://www.ebi.ac.uk/ena/browser/home /
ERR3148200	Mexico	YES	resistant	Available at ENA portals https://www.ebi.ac.uk/ena/browser/home
ERR3148201	Mexico	No	resistant	Available at ENA portals https://www.ebi.ac.uk/ena/browser/home /
ERR3148202	Mexico	No	resistant	Available at ENA portals https://www.ebi.ac.uk/ena/browser/home
ERR3148203	Mexico	No	resistant	Available at ENA portals https://www.ebi.ac.uk/ena/browser/home /
ERR3148204	Mexico	No	resistant	Available at ENA portals https://www.ebi.ac.uk/ena/browser/home
ERR3148205	Mexico	No	resistant	Available at ENA portals https://www.ebi.ac.uk/ena/browser/home /
ERR3148207	Mexico	YES	sensitive	Available at ENA portals https://www.ebi.ac.uk/ena/browser/home /
ERR3148208	Mexico	YES	resistant	Available at ENA portals https://www.ebi.ac.uk/ena/browser/home
ERR3148209	Mexico	YES	sensitive	Available at ENA portals https://www.ebi.ac.uk/ena/browser/home /
ERR3148210	Mexico	No	sensitive	Available at ENA portals https://www.ebi.ac.uk/ena/browser/home
ERR3148211	Mexico	YES	resistant	Available at ENA portals https://www.ebi.ac.uk/ena/browser/home /

ERR3148212	Mexico	No	sensitive	Available at ENA portals https://www.ebi.ac.uk/ena/browser/home
ERR3148213	Mexico	No	resistant	Available at ENA portals https://www.ebi.ac.uk/ena/browser/home /
ERR3148216	Mexico	YES	resistant	Available at ENA portals https://www.ebi.ac.uk/ena/browser/home
ERR3148217	Mexico	YES	resistant	Available at ENA portals https://www.ebi.ac.uk/ena/browser/home /
ERR3148218	Mexico	No	sensitive	Available at ENA portals https://www.ebi.ac.uk/ena/browser/home
ERR3148220	Mexico	No	sensitive	Available at ENA portals https://www.ebi.ac.uk/ena/browser/home /
ERR3148222	Mexico	YES	resistant	Available at ENA portals https://www.ebi.ac.uk/ena/browser/home
ERR3148224	Mexico	No	sensitive	Available at ENA portals https://www.ebi.ac.uk/ena/browser/home /
ERR3148225	Mexico	No	resistant	Available at ENA portals https://www.ebi.ac.uk/ena/browser/home /
ERR3148226	Mexico	YES	sensitive	Available at ENA portals https://www.ebi.ac.uk/ena/browser/home
ERR3148229	Mexico	No	sensitive	Available at ENA portals https://www.ebi.ac.uk/ena/browser/home /
SRR10379888	Moldova	No	sensitive	Available at TB portals https://tbportals.niaid.nih.gov/
SRR10379892	Moldova	No	resistant	Available at TB portals https://tbportals.niaid.nih.gov/
SRR10379896	Moldova	No	resistant	Available at TB portals https://tbportals.niaid.nih.gov/
SRR10379899	Moldova	No	resistant	Available at TB portals https://tbportals.niaid.nih.gov/
SRR10379901	Moldova	No	sensitive	Available at TB portals https://tbportals.niaid.nih.gov/
SRR10379903	Moldova	No	resistant	Available at TB portals https://tbportals.niaid.nih.gov/
SRR10379906	Moldova	No	resistant	Available at TB portals https://tbportals.niaid.nih.gov/
SRR10379908	Moldova	No	resistant	Available at TB portals https://tbportals.niaid.nih.gov/
SRR10379912	Moldova	No	resistant	Available at TB portals https://tbportals.niaid.nih.gov/
SRR10379915	Moldova	No	sensitive	Available at TB portals https://tbportals.niaid.nih.gov/
SRR10379917	Moldova	No	resistant	Available at TB portals https://tbportals.niaid.nih.gov/
SRR10379932	Moldova	No	resistant	Available at TB portals https://tbportals.niaid.nih.gov/
SRR10379936	Moldova	No	resistant	Available at TB portals https://tbportals.niaid.nih.gov/

SRR10379938	Moldova	No	sensitive	Available at TB portals https://tbportals.niaid.nih.gov/
SRR10379944	Moldova	No	resistant	Available at TB portals https://tbportals.niaid.nih.gov/
SRR10379945	Moldova	YES	resistant	Available at TB portals https://tbportals.niaid.nih.gov/
SRR10379971	Moldova	No	resistant	Available at TB portals https://tbportals.niaid.nih.gov/
SRR10379987	Moldova	No	resistant	Available at TB portals https://tbportals.niaid.nih.gov/
SRR10379988	Moldova	No	sensitive	Available at TB portals https://tbportals.niaid.nih.gov/
SRR10379990	Moldova	YES	resistant	Available at TB portals https://tbportals.niaid.nih.gov/
SRR10380002	Moldova	No	resistant	Available at TB portals https://tbportals.niaid.nih.gov/
SRR10380006	Moldova	No	sensitive	Available at TB portals https://tbportals.niaid.nih.gov/
SRR10380009	Moldova	No	resistant	Available at TB portals https://tbportals.niaid.nih.gov/
SRR10380011	Moldova	No	resistant	Available at TB portals https://tbportals.niaid.nih.gov/
SRR10380014	Moldova	No	resistant	Available at TB portals https://tbportals.niaid.nih.gov/
SRR10380016	Moldova	No	sensitive	Available at TB portals https://tbportals.niaid.nih.gov/
SRR10380027	Moldova	No	sensitive	Available at TB portals https://tbportals.niaid.nih.gov/
SRR10380037	Moldova	No	resistant	Available at TB portals https://tbportals.niaid.nih.gov/
SRR10380039	Moldova	No	resistant	Available at TB portals https://tbportals.niaid.nih.gov/
SRR10380043	Moldova	No	resistant	Available at TB portals https://tbportals.niaid.nih.gov/
SRR10380047	Moldova	YES	resistant	Available at TB portals https://tbportals.niaid.nih.gov/
SRR10380057	Moldova	YES	resistant	Available at TB portals https://tbportals.niaid.nih.gov/
SRR10380076	Moldova	YES	resistant	Available at TB portals https://tbportals.niaid.nih.gov/
SRR10380130	Moldova	No	resistant	Available at TB portals https://tbportals.niaid.nih.gov/
SRR10380154	Moldova	No	sensitive	Available at TB portals https://tbportals.niaid.nih.gov/
SRR10380160	Moldova	No	resistant	Available at TB portals https://tbportals.niaid.nih.gov/
SRR10380163	Moldova	No	sensitive	Available at TB portals https://tbportals.niaid.nih.gov/
SRR10380175	Moldova	YES	sensitive	Available at TB portals https://tbportals.niaid.nih.gov/
SRR10380179	Moldova	No	sensitive	Available at TB portals https://tbportals.niaid.nih.gov/
SRR10380193	Moldova	No	sensitive	Available at TB portals https://tbportals.niaid.nih.gov/
SRR10380195	Moldova	No	sensitive	Available at TB portals https://tbportals.niaid.nih.gov/
SRR10380211	Moldova	YES	sensitive	Available at TB portals https://tbportals.niaid.nih.gov/
SRR10380212	Moldova	No	sensitive	Available at TB portals https://tbportals.niaid.nih.gov/

SRR10380213	Moldova	No	resistant	Available at TB portals https://tbportals.niaid.nih.gov/
SRR10380215	Moldova	No	resistant	Available at TB portals https://tbportals.niaid.nih.gov/
SRR10380216	Moldova	No	resistant	Available at TB portals https://tbportals.niaid.nih.gov/
SRR10380217	Moldova	No	resistant	Available at TB portals https://tbportals.niaid.nih.gov/
SRR10380219	Moldova	No	sensitive	Available at TB portals https://tbportals.niaid.nih.gov/
SRR10380221	Moldova	No	sensitive	Available at TB portals https://tbportals.niaid.nih.gov/
SRR10380232	Moldova	No	sensitive	Available at TB portals https://tbportals.niaid.nih.gov/
SRR10380235	Moldova	No	sensitive	Available at TB portals https://tbportals.niaid.nih.gov/
SRR10380236	Moldova	No	sensitive	Available at TB portals https://tbportals.niaid.nih.gov/
SRR10380240	Moldova	No	resistant	Available at TB portals https://tbportals.niaid.nih.gov/
SRR3743493	Moldova	YES	sensitive	Available at TB portals https://tbportals.niaid.nih.gov/
SRR5153902	Moldova	YES	resistant	Available at TB portals https://tbportals.niaid.nih.gov/
SRR10808334	Peru	YES	sensitive	Available at ENA portals https://www.ebi.ac.uk/ena/browser/home /
SRR10808337	Peru	YES	resistant	Available at ENA portals https://www.ebi.ac.uk/ena/browser/home
SRR10808342	Peru	YES	sensitive	Available at ENA portals https://www.ebi.ac.uk/ena/browser/home /
SRR10808344	Peru	YES	sensitive	Available at ENA portals https://www.ebi.ac.uk/ena/browser/home
SRR10808353	Peru	YES	resistant	Available at ENA portals https://www.ebi.ac.uk/ena/browser/home /
SRR10808370	Peru	YES	resistant	Available at ENA portals https://www.ebi.ac.uk/ena/browser/home
SRR10808409	Peru	YES	resistant	Available at ENA portals https://www.ebi.ac.uk/ena/browser/home /
SRR10808462	Peru	YES	sensitive	Available at ENA portals https://www.ebi.ac.uk/ena/browser/home /
SRR10808467	Peru	YES	sensitive	Available at ENA portals https://www.ebi.ac.uk/ena/browser/home
SRR10808472	Peru	YES	sensitive	Available at ENA portals https://www.ebi.ac.uk/ena/browser/home /
SRR10808520	Peru	YES	resistant	Available at ENA portals https://www.ebi.ac.uk/ena/browser/home
SRR10808536	Peru	YES	sensitive	Available at ENA portals https://www.ebi.ac.uk/ena/browser/home /

SRR10808543	Peru	YES	sensitive	Available at ENA portals https://www.ebi.ac.uk/ena/browser/home
SRR10808572	Peru	YES	resistant	Available at ENA portals https://www.ebi.ac.uk/ena/browser/home /
SRR10808574	Peru	YES	resistant	Available at ENA portals https://www.ebi.ac.uk/ena/browser/home
SRR10808607	Peru	YES	sensitive	Available at ENA portals https://www.ebi.ac.uk/ena/browser/home /
SRR10808641	Peru	YES	sensitive	Available at ENA portals https://www.ebi.ac.uk/ena/browser/home
SRR10808654	Peru	YES	sensitive	Available at ENA portals https://www.ebi.ac.uk/ena/browser/home /
SRR10808670	Peru	YES	resistant	Available at ENA portals https://www.ebi.ac.uk/ena/browser/home
SRR10808688	Peru	YES	resistant	Available at ENA portals https://www.ebi.ac.uk/ena/browser/home /
SRR10808704	Peru	YES	resistant	Available at TB portals https://tbportals.niaid.nih.gov/
SRR10808810	Peru	YES	sensitive	Available at ENA portals https://www.ebi.ac.uk/ena/browser/home /
SRR10808827	Peru	YES	sensitive	Available at ENA portals https://www.ebi.ac.uk/ena/browser/home
SRR10808882	Peru	YES	sensitive	Available at ENA portals https://www.ebi.ac.uk/ena/browser/home /
SRR10808917	Peru	YES	resistant	Available at ENA portals https://www.ebi.ac.uk/ena/browser/home
SRR10808924	Peru	YES	sensitive	Available at ENA portals https://www.ebi.ac.uk/ena/browser/home /
SRR10808926	Peru	YES	sensitive	Available at ENA portals https://www.ebi.ac.uk/ena/browser/home /
SRR10808928	Peru	YES	sensitive	Available at ENA portals https://www.ebi.ac.uk/ena/browser/home
SRR10808948	Peru	YES	resistant	Available at ENA portals https://www.ebi.ac.uk/ena/browser/home /
SRR10525318	Romania	No	resistant	Available at TB portals https://tbportals.niaid.nih.gov/
SRR10525325	Romania	No	resistant	Available at TB portals https://tbportals.niaid.nih.gov/
SRR10525360	Romania	YES	resistant	Available at TB portals https://tbportals.niaid.nih.gov/
SRR10525371	Romania	No	resistant	Available at TB portals https://tbportals.niaid.nih.gov/

SRR3544723	Romania	No	sensitive	Available at TB portals https://tbportals.niaid.nih.gov/
SRR3743199	Romania	YES	sensitive	Available at TB portals https://tbportals.niaid.nih.gov/
SRR5486869	Romania	YES	resistant	Available at TB portals https://tbportals.niaid.nih.gov/
SRR5486894	Romania	No	resistant	Available at TB portals https://tbportals.niaid.nih.gov/
SRR5486901	Romania	No	resistant	Available at TB portals https://tbportals.niaid.nih.gov/
SRR7592322	Romania	No	resistant	Available at TB portals https://tbportals.niaid.nih.gov/
SRR7592333	Romania	No	resistant	Available at TB portals https://tbportals.niaid.nih.gov/
SRR7592379	Romania	No	resistant	Available at TB portals https://tbportals.niaid.nih.gov/
SRR9738478	Romania	No	sensitive	Available at TB portals https://tbportals.niaid.nih.gov/
SRR9738483	Romania	No	resistant	Available at TB portals https://tbportals.niaid.nih.gov/
SRR9738491	Romania	No	sensitive	Available at TB portals https://tbportals.niaid.nih.gov/
SRR9738494	Romania	No	resistant	Available at TB portals https://tbportals.niaid.nih.gov/
SRR9738495	Romania	YES	resistant	Available at TB portals https://tbportals.niaid.nih.gov/
SRR9738514	Romania	No	resistant	Available at TB portals https://tbportals.niaid.nih.gov/
SRR9738528	Romania	No	resistant	Available at TB portals https://tbportals.niaid.nih.gov/
SRR9738545	Romania	No	resistant	Available at TB portals https://tbportals.niaid.nih.gov/
SRR9738552	Romania	No	resistant	Available at TB portals https://tbportals.niaid.nih.gov/
G1036	Spain	YES	sensitive	Available at ENA portals https://www.ebi.ac.uk/ena/browser/home /
G1107	Spain	YES	sensitive	Available at ENA portals https://www.ebi.ac.uk/ena/browser/home
G1243	Spain	YES	sensitive	Available at ENA portals https://www.ebi.ac.uk/ena/browser/home /
G1302m	Spain	YES	sensitive	Available at ENA portals https://www.ebi.ac.uk/ena/browser/home
G1303	Spain	YES	sensitive	Available at ENA portals https://www.ebi.ac.uk/ena/browser/home /
G1304	Spain	YES	sensitive	Available at ENA portals https://www.ebi.ac.uk/ena/browser/home
G1312	Spain	YES	resistant	Available at ENA portals https://www.ebi.ac.uk/ena/browser/home /
G1320	Spain	YES	sensitive	Available at ENA portals https://www.ebi.ac.uk/ena/browser/home

G1327	Spain	YES	sensitive	Available at ENA portals https://www.ebi.ac.uk/ena/browser/home /
G1359	Spain	YES	sensitive	Available at ENA portals https://www.ebi.ac.uk/ena/browser/home /
G1449	Spain	YES	sensitive	Available at ENA portals https://www.ebi.ac.uk/ena/browser/home /
G1509	Spain	YES	sensitive	Available at ENA portals https://www.ebi.ac.uk/ena/browser/home
G1521	Spain	YES	resistant	Available at ENA portals https://www.ebi.ac.uk/ena/browser/home /
G1523	Spain	YES	resistant	Available at ENA portals https://www.ebi.ac.uk/ena/browser/home
G1532	Spain	YES	sensitive	Available at ENA portals https://www.ebi.ac.uk/ena/browser/home /
G1533	Spain	YES	sensitive	Available at ENA portals https://www.ebi.ac.uk/ena/browser/home
G1568	Spain	YES	sensitive	Available at ENA portals https://www.ebi.ac.uk/ena/browser/home /
G1573	Spain	YES	resistant	Available at ENA portals https://www.ebi.ac.uk/ena/browser/home
G1583	Spain	YES	sensitive	Available at ENA portals https://www.ebi.ac.uk/ena/browser/home /
G1617	Spain	YES	sensitive	Available at ENA portals https://www.ebi.ac.uk/ena/browser/home /
G1657	Spain	YES	resistant	Available at ENA portals https://www.ebi.ac.uk/ena/browser/home /
G1672	Spain	YES	resistant	Available at ENA portals https://www.ebi.ac.uk/ena/browser/home
G1684	Spain	YES	sensitive	Available at ENA portals https://www.ebi.ac.uk/ena/browser/home /
G1728	Spain	YES	sensitive	Available at ENA portals https://www.ebi.ac.uk/ena/browser/home
G1731	Spain	YES	sensitive	Available at ENA portals https://www.ebi.ac.uk/ena/browser/home /
G1763	Spain	YES	sensitive	Available at ENA portals https://www.ebi.ac.uk/ena/browser/home
G1764	Spain	YES	sensitive	Available at ENA portals https://www.ebi.ac.uk/ena/browser/home /

G1765	Spain	YES	sensitive	Available at ENA portals https://www.ebi.ac.uk/ena/browser/home
G1766	Spain	YES	sensitive	Available at ENA portals https://www.ebi.ac.uk/ena/browser/home /
G1782	Spain	YES	sensitive	Available at ENA portals https://www.ebi.ac.uk/ena/browser/home
G1792	Spain	YES	sensitive	Available at ENA portals https://www.ebi.ac.uk/ena/browser/home /
G1910	Spain	YES	sensitive	Available at ENA portals https://www.ebi.ac.uk/ena/browser/home
G1934	Spain	YES	resistant	Available at ENA portals https://www.ebi.ac.uk/ena/browser/home /
G271	Spain	YES	resistant	Available at ENA portals https://www.ebi.ac.uk/ena/browser/home
G357m	Spain	YES	sensitive	Available at ENA portals https://www.ebi.ac.uk/ena/browser/home /
